# Elevated NPM1 and FBL expression correlates with prostate cancer aggressiveness and progression

**DOI:** 10.1002/path.6447

**Published:** 2025-07-24

**Authors:** Sarina Saffarian, Ziwei Cai, Jordan Lam, Htoo Zarni Oo, Syam Somasekharan

**Affiliations:** ^1^ Vancouver Prostate Centre, Department of Urologic Sciences University of British Columbia Vancouver BC Canada

**Keywords:** prostate cancer, castration‐resistant prostate cancer, cancer progression, nucleolus, nucleophosmin, NPM1, fibrillarin, FBL, migration, invasion

## Abstract

The nucleolus is a membrane‐less body present in the nucleus of the cell. The nucleolus is mainly involved in ribosomal RNA (rRNA) transcription and ribosome biogenesis for protein translation. During cancer formation, nucleolar morphology is altered, and many nucleolar proteins are expressed at a higher level, leading to enhanced ribosome biogenesis and protein translation, which supports cancer aggressiveness, proliferation, migration, and invasion. In this study, we investigated the association of two nucleolar proteins, nucleophosmin (NPM1), and fibrillarin (FBL), with prostate cancer (PCa) aggressiveness and progression. We investigated their cellular localization and expression in different PCa patient tissue specimens and their role in regulating proliferation, migration, invasion, and nucleolar morphology. Our results indicate that NPM1 and FBL are present in the nucleolus of both PCa and noncancerous prostatic cells. The expression of NPM1 and FBL was enhanced in aggressive castration‐resistant PCa (CRPC) and neuro‐endocrine PCa (NEPC) patient specimens compared to hormone‐naïve PCa (HNPC) patient specimens. The expression of NPM1 was enhanced in high‐Gleason score PCa compared to low‐Gleason score PCa. Silencing of NPM1 and FBL significantly reduced the proliferation, migration, and invasion of PCa cells without affecting noncancerous prostatic cells. Silencing of NPM1 and FBL also changed the morphology of nucleoli in both PCa and noncancerous prostatic cells, where NPM1 silencing fragmented the nucleoli and FBL silencing condensed the nucleoli. Our results suggest that NPM1 and FBL expression correlates with PCa aggressiveness and PCa cells may exhibit a unique dependence on NPM1 or FBL for PCa progression. © 2025 The Author(s). *The Journal of Pathology* published by John Wiley & Sons Ltd on behalf of The Pathological Society of Great Britain and Ireland.

## Introduction

Prostate cancer (PCa) is the second most frequent cancer diagnosis made in men, with a global death rate of 0.4 million every year [1]. Based on its growth properties, PCa is broadly divided into indolent and aggressive categories [2]. Indolent cancer is slow‐growing, and patients are advised for active surveillance to combat overtreatment by delaying or avoiding unnecessary definitive treatment and its associated morbidity [3]. Aggressive cancer is fast‐growing, and immediate intervention with surgery and/or radiation is required [4, 5]. A significant number of patients receive androgen deprivation therapy (ADT) that decreases the level of androgens and reduces cancer growth [6]. Although ADT initially reduces cancer growth, the relapse of cancer is inevitable [7]. The relapsed cancer types, castration‐resistant PCa (CRPC) and neuroendocrine PCa (NEPC), are aggressive forms with highly invasive and metastatic behaviors [8]. Most patients with CRPC and NEPC die within 2 years of relapse [9]. Detection procedures distinguishing aggressive from indolent cancers are not well standardized due to the absence of definitive biomarkers. This delays early detection of aggressive cancer and timely therapeutic intervention. Therefore, there is an urgent need to identify new biomarkers and therapeutic targets to enhance the detection and management of aggressive forms of PCa.

The nucleolus, a membrane‐less structure within the nucleus, is primarily involved in ribosome biogenesis [10, 11, 12, 13]. Ribosome biogenesis is a complex process and occurs in three specialized nucleolar regions, the fibrillar center (FC), dense fibrillar center (DFC), and granular center (GC), each contributing to different stages of the ribosome production [13]. The transcription of ribosomal DNA (rDNA) to ribosomal RNA (rRNA) takes place in the FC. Following their production, these rRNAs undergo a series of splicing and modification steps in the DFC. They are then assembled with ribosomal proteins to form preribosomal particles in the GC. Following ribosome assembly, the ribosomal particles are exported to the cytoplasm, where they mature into fully functional ribosomes and participate in mRNA translation. The close relationship between nucleolar organization and ribosome biogenesis highlights that any disruption in nucleolar structure and activity can impact ribosome production and protein translation [13].

Nucleolar morphology and functions are altered in cancer cells. Cancer cells have irregular and enlarged nucleoli compared to noncancerous cells [14, 15, 16]. The enlarged nucleoli in cancer cells are associated with enhanced nucleolar activity with increased ribosome production, protein synthesis, cellular proliferation, migration, and invasion [17, 18]. In PCa, nucleolar enlargement and increased numbers of nucleoli are some of the earliest morphological changes associated with developing premalignant prostate intraepithelial neoplasia (PIN) lesions and aggressive adenocarcinomas [19, 20]. The altered nucleolar morphology and activity are significantly regulated through nucleolar proteins. During the progression of cancer, the expression of many nucleolar proteins is upregulated, leading to changes in nucleolar morphology and nucleolar activity [16, 21, 22].

In this study, we selected nucleophosmin (NPM1) present in the GC region [23, 24] and fibrillarin (FBL) present in the DFC region [24, 25, 26] of the nucleolus and analyzed their cellular localization, expression in PCa patient specimens, and role in regulating proliferation, migration and invasion, and nucleolar morphology. Our results indicate that NPM1 and FBL are localized in the nucleolus of both PCa and noncancerous prostatic cells. The expression of NPM1 and FBL was enhanced in CRPC and NEPC patient specimens compared to patients that are hormone naïve (HNPC) or subjected to neoadjuvant hormone therapy (NHT). The expression of NPM1 was also increased in high‐Gleason score compared to low‐Gleason score PCa specimens. Silencing of NPM1 and FBL significantly reduced the proliferation, migration, and invasion of PCa cells without affecting noncancerous prostatic cells. Silencing of NPM1 induced the fragmentation of nucleoli, while silencing of FBL induced the condensation of nucleoli in both PCa and noncancerous prostatic cells. Our results indicate that the expression of NPM1 and FBL are associated with PCa aggressiveness, and they support the proliferation, migration, and invasion of PCa cells.

## Materials and methods

### Antibodies, cell lines, and reagents

FBL (sc‐374022) and NPM1 (sc‐47725) (Santa Cruz Biotechnology, Dallas, TX, USA); fluorescent secondary antibodies [mouse, Alexa Fluor 488 (A‐11059)/594 (A‐11062) (Thermo Fisher Scientific, Waltham, MA, USA)]; RPMI (11875093), FBS (A5256701), penicillin–streptomycin (15070063), and RNAiMAX transfection (13778075) (Thermo Fisher Scientific); FluorSave (345789) (Merck, Rahway, NJ, USA). Silver nitrate (204390) and bovine gelatin (G9391) (Sigma‐Aldrich, St. Louis, MO, USA). LNCaP, LNCaP C4‐2, 22Rv1, PC3, PNT1B, and BPH‐1 (ATCC, Manassas, VA, USA). The cells were cultured in RPMI 1640, supplemented with 10% FBS and 1% penicillin–streptomycin, under standard conditions (37 °C, 95% humidity, and 5% CO₂). siRNAs targeting human NPM1 and FBL [NPM1 (sc‐29771) and FBL (sc‐37883)] (Santa Cruz Biotechnology); nontargeting control siRNA (CTM‐1005912) (Dharmacon, Lafayette, CO, USA).

### Quantification of NPM1 and FBL in PCa patient specimens

Tissue microarray (TMA) core tumor regions were imaged uniformly and assigned unique identifiers to correlate nucleolar intensity with clinical parameters. Arbitrary numbers were assigned to TMA cores based on visual assessment of differences in the intensity of antibody staining for NPM1 and FBL proteins in the nucleoli of tumor cells within each core. To validate the accuracy of the grouping, the nucleolar intensity was measured using the ‘Integrated Density’ function in ImageJ (National Institutes of Health, Bethesda, MD, USA). This ensured that intensity levels within each group were consistent and the scoring method was reliable. Statistical analyses, including Student's *t*‐test and one‐way ANOVA, were performed using GraphPad Prism (version 10.4.1) (Boston, MA, USA). Statistical significance was defined as *p < 0.05, **p < 0.01, ***p < 0.001, and ****p < 0.0001.

### Immunofluorescence

Cells seeded at 20%–25% confluence in 6‐cm culture dishes containing round cover glasses (12CIR‐1D; Thermo Fisher Scientific) were subjected to immunofluorescence (IF) as described previously [27]. Cells were fixed in 4% paraformaldehyde (PFA) for 20 min and permeabilized with PBS‐T (0.05% Triton X‐100 in PBS) for 20 min. The cells were then blocked for 30 min in PBS‐T containing 5% BSA and incubated with primary antibodies (1:100) for 1 h in PBS containing 2.5% BSA. Cells were washed in PBS‐T for 30 min (3 × 10 min), followed by incubation with secondary antibodies (1:200) in PBS‐T containing 2.5% BSA for 1 h. Cells were then washed in PBS‐T for 30 min (3 × 10 min). The IF slides were immersed in DAPI (10 μm) for nuclear staining, mounted with FluorSave (Merck), and viewed using ZEISS LSM780, confocal microscope (ZEISS, Oberkochen, Baden‐Württemberg, Germany) with ×60 and ×100 oil‐immersion objective lenses. Images were captured using ZEN imaging software (ZEISS).

### Immunohistochemistry (IHC)

TMAs (Pathology core, Vancouver Prostate Centre, Vancouver, British Columbia, Canada) were processed for IHC using a protocol described previously [28, 29, 30]. In brief, the TMA slides were subjected to deparaffinization and activation of antigen epitopes using a Ventana BenchMark ULTRA Slide Stainer system (Roche, Basel, Switzerland) and staining using antibodies against NPM1 and FBL. Sections were counterstained with hematoxylin and viewed using a light microscope (Zeiss Axio Observer Z1; ZEISS) with ×10 and ×40 objective lenses. Images were captured using ZEN imaging software. A board‐certified pathologist scored the staining for each antibody. Tukey's multiple‐comparisons analysis was performed to assess the expressions of each protein.

### Proliferation assay

The Incucyte S3 Live‐Cell Analysis System (Sartorius, MI, USA) was used to monitor cell proliferation and analyze cell confluence. Reverse‐transfected cells were seeded at a density of 1 × 10^4^ cells per well in 96‐well plates. Cells were incubated and monitored under standard conditions (37 °C, 95% humidity, and 5% CO₂) for 96 h after transfection.

### Transwell invasion and migration assay

For the Transwell invasion assay, 20 μl of thawed Corning Matrigel was diluted in 1 ml of serum‐free RPMI medium, and 150 μl of this mixture was applied to the upper surface of each Transwell membrane, followed by incubation for 1 h at 37 °C. For the migration assay, no Matrigel was applied. The lower chamber was filled with RPMI medium containing 10% FBS to serve as a chemoattractant. The cells were harvested 48 h after transfection with siRNAs targeting human NPM1, FBL, or nontargeting control siRNA and then resuspended in serum‐free RPMI. Cells were seeded at a density of 7.5 × 10^3^ cells per well in Corning Costar 24‐well plates and incubated under standard conditions (37 °C, 95% humidity, and 5% CO₂) for 24 h. Following incubation, cells were fixed with 4% PFA and stained with 0.1% crystal violet for 20 min. Images of representative fields were captured, and cell confluence was quantified using SnapCyte software (SnapCyte, Vancouver, BC, Canada). Each condition was tested in triplicate.

### Silver nitrate staining

The coverslips containing cells were washed in PBS and incubated with PBS‐T (0.05% Tween 20 in PBS) for 30 min at room temperature to permeabilize cell membranes and allow silver nitrate to react with nucleolar organizer regions (AgNORs). Following PBS‐T treatment, coverslips were thoroughly washed with distilled water to prevent any reaction with silver nitrate solution. Next, the staining solution was prepared by mixing two parts of 50% w/v concentrated solution of silver nitrate with one part of 2% w/v gelatin in 1% formic acid (pH 3), immediately before use, as it is light‐sensitive and degrades quickly. The freshly mixed solution, which should appear clear, was applied to coverslips and incubated in the dark for 25 min, during which the solution gradually turned dark brown. After incubation, the solution was aspirated, and coverslips were washed thoroughly with distilled water. The reaction was then blocked by incubating coverslips in 5% acetic acid in the dark for 10 min. Coverslips were subsequently mounted on slides, dried, stored in a dark environment, and scanned.

## Results

### Expression and localization of nucleolar proteins

**Figure 1:**
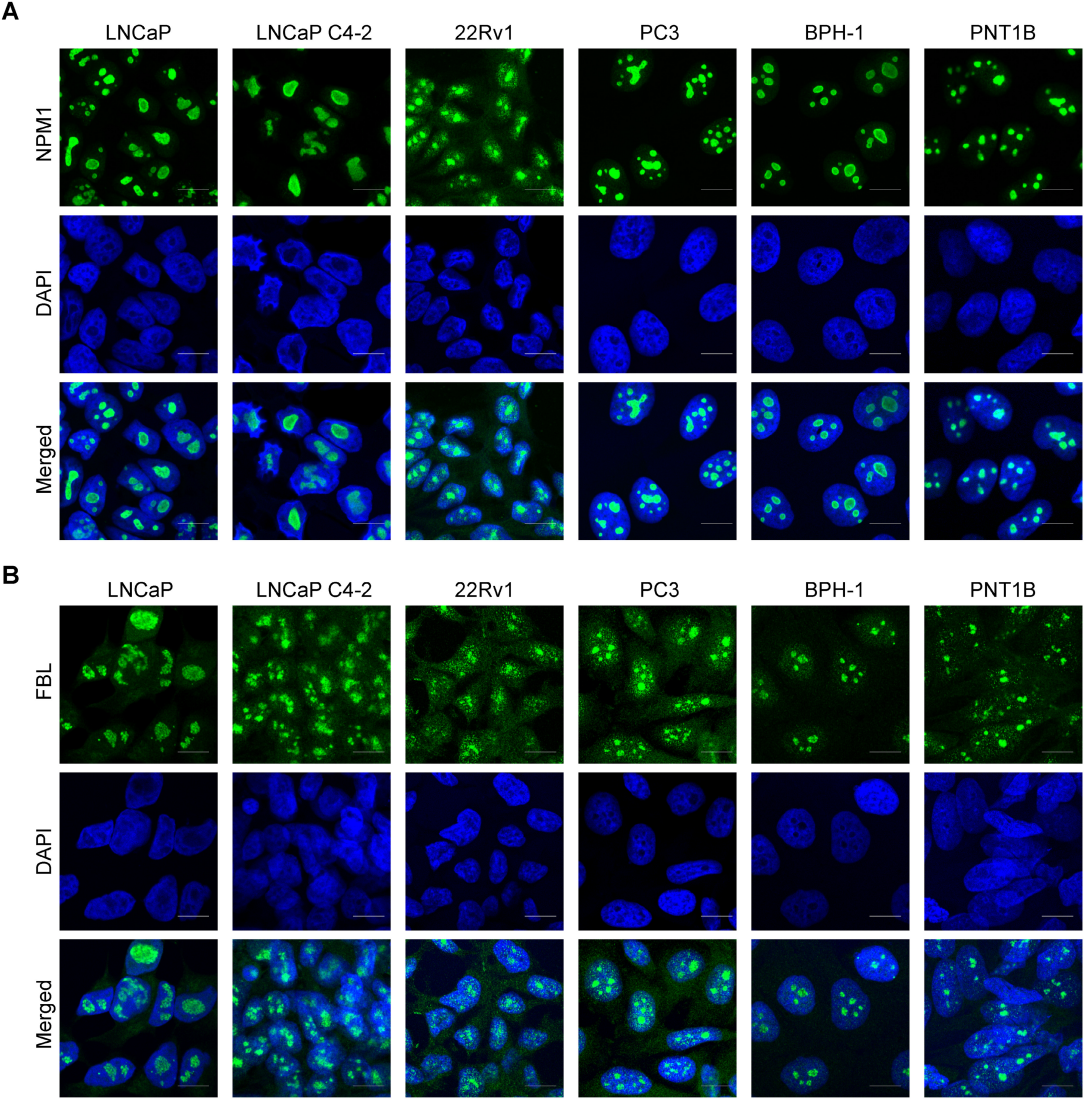
NPM1 and FBL localize in the nucleolus. Immunofluorescent images showing the localization of NPM1 (A) and FBL (B) in a panel of PCa cell lines, LNCaP, LNCaP C4‐2, 22Rv1, and PC3, and noncancerous prostatic cell lines, BPH‐1 and PNT1B. Scale bar, 10 μm.

We used immunofluorescence with validated antibodies to check the localization and staining pattern of nucleolar proteins NPM1 and FBL in a panel of PCa and noncancerous prostatic cells. The cancerous cell lines used were androgen‐sensitive LNCaP [31], androgen‐independent PC3 [32], castration‐resistant LNCaP C4‐2 [33] and 22Rv1 [34], and noncancerous cell lines used were BPH‐1 [35] and PNT1B [36]. We found that both NPM1 and FBL were expressed in the nucleoli of both cancerous and noncancerous cell lines. The NPM1 that stains the GC and FBL that stains the DFC of the nucleolus showed a differential staining pattern between the tested cell lines. The NPM1 stained an enlarged single GC region in LNCaP and LNCaP C4‐2 cells (Figure 1A), while multiple GC regions were observed with NPM1 staining in 22Rv1, PC3, BPH‐1, and PNT1B cells (Figure 1A). Similarly, the FBL stained a larger area of the DFC in LNCaP and LNCaP C4‐2 cells (Figure 1B). On the other hand, FBL stained multiple nucleoli in 22Rv1, PC3, BPH‐1, and PNT1B cells (Figure 1B). The difference in the staining patterns of NPM1 and FBL indicates the diversity in the distribution of these proteins in the GC and DFC regions in different cell lines.

### Differential expression of NPM1 and FBL in HNPC, NHT, CRPC, and NEPC patient specimens

**Figure 2:**
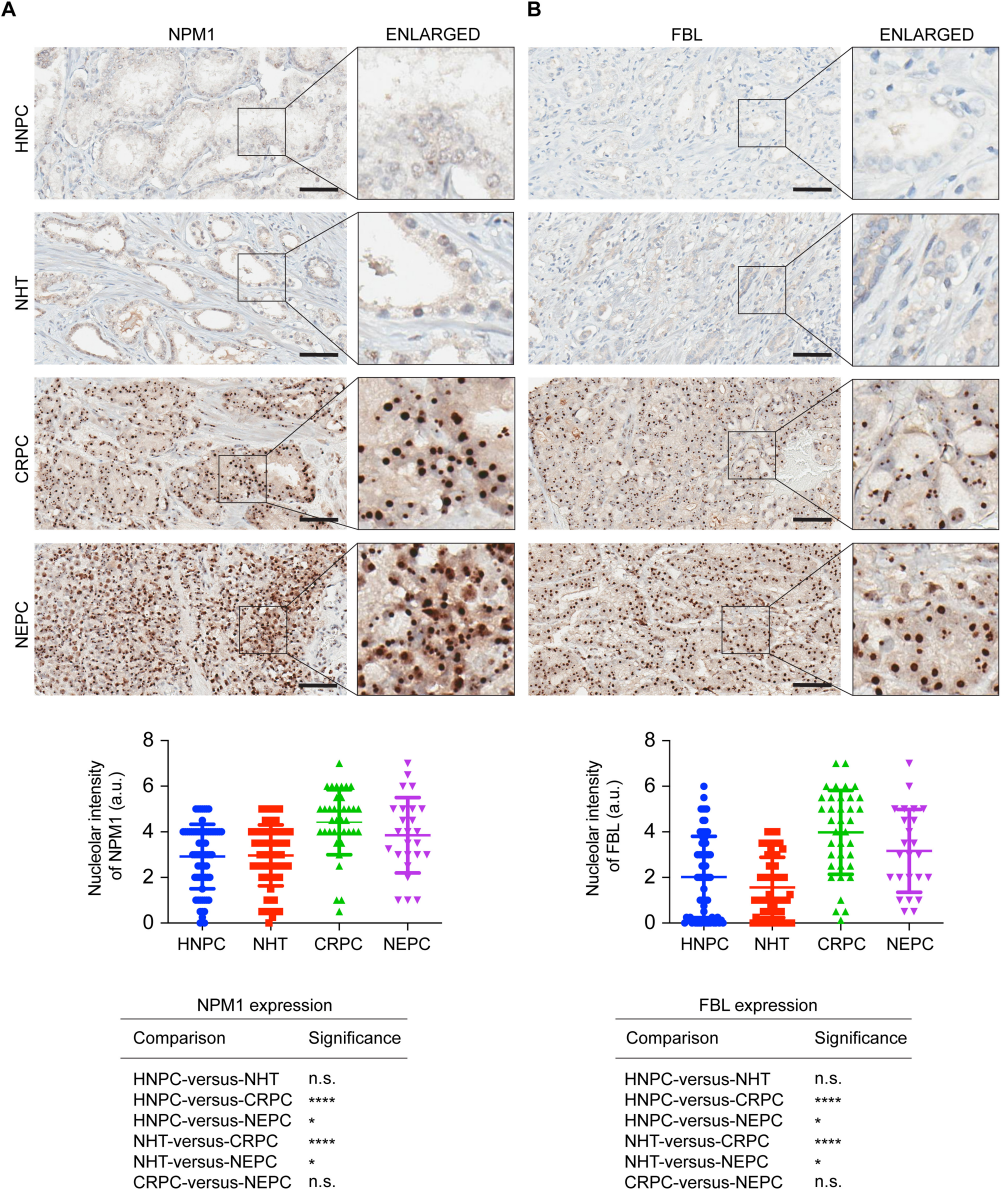
Expression of NPM1 and FBL is enhanced in CRPC and NEPC patient specimens. IHC staining of NPM1 (A) and FBL (B) in HNPC, NHT, CRPC, and NEPC patient specimens. Scale bar, 60 μm. A part of the image is enlarged and shown on the right side. Bottom panels show the quantification of IHC staining of NPM1 and FBL. The significance between different conditions is shown in tables. *p < 0.05; ****p < 0.0001; n.s., not significant.

The mRNA expression of *NPM1* and *FBL* was increased in PCa specimens compared to normal prostatic tissues in the prostate adenocarcinoma [The Cancer Genome Atlas (TCGA)] dataset accessed through UALCAN (The University of ALabama at Birmingham CANcer data analysis Portal) [37] (supplementary material, Figure S1A,B). We further analyzed the protein expression of NPM1 and FBL in a large cohort of 600 patient specimens by IHC using validated antibodies against individual proteins. The cohort contained specimens of benign prostatic hyperplasia (BPH) [38], HNPC [39], NHT [40], CRPC [41], and NEPC [42]. The IHC stained slides were scanned and subjected to intensity quantification and analysis of individual proteins in the nucleolus. The expression of NPM1 and FBL was absent in BPH specimens (supplementary material, Figure S2). While comparing the intensity of staining between the HNPC, NHT, CRPC, and NEPC specimens, we found that the expression of NPM1 was significantly enhanced in CRPC and NEPC compared to HNPC and NHT specimens (Figure 2A). The expression of NPM1 did not change between HNPC and NHT or CRPC and NEPC specimens (Figure 2A). The expression of FBL was also significantly enhanced in CRPC and NEPC compared to HNPC and NHT specimens (Figure 2B). Similar to NPM1, FBL expression was unchanged between HNPC and NHT or CRPC and NEPC specimens (Figure 2B). These results indicate that NPM1 and FBL expression are predictors of aggressive forms of PCa as their expression is enhanced in CRPC and NEPC.

### Expression of NPM1 and FBL in different Gleason score PCa patient specimens

**Figure 3:**
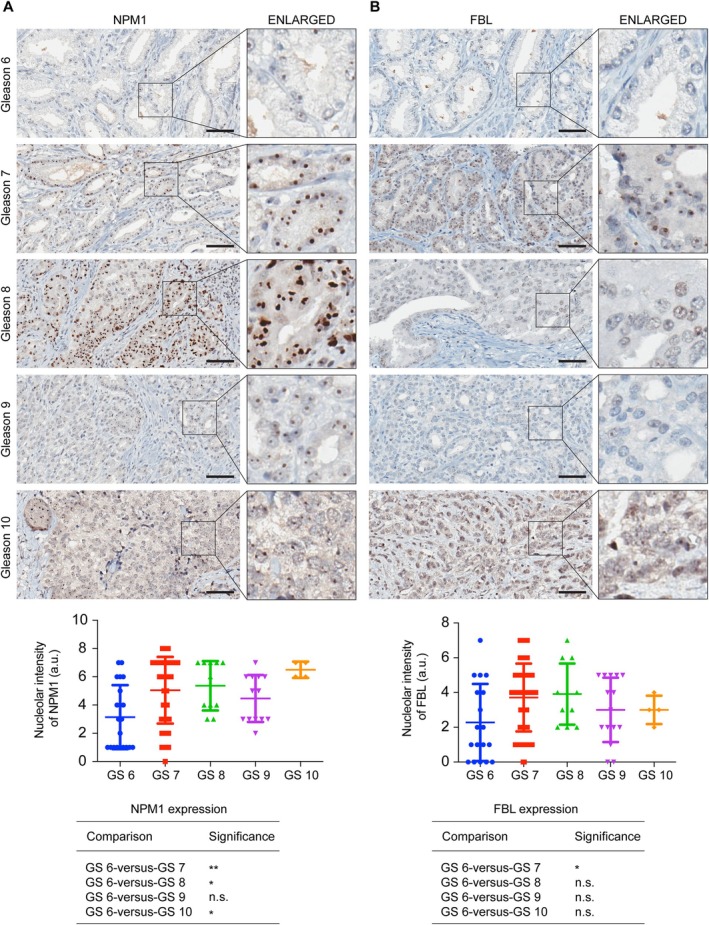
Expression of NPM1 and FBL in different Gleason score PCa patient specimens. IHC staining of NPM1 (A) and FBL (B) in different Gleason score (Gleason 6–10) PCa patient specimens. Scale bar, 60 μm. A part of the image is enlarged and shown on the right side. Bottom panels show the quantification of IHC staining of NPM1 and FBL. The significance between different conditions is shown in tables. *p < 0.05; **p < 0.01; n.s., not significant.

As the expression of NPM1 and FBL was found to be enhanced in CRPC and NEPC specimens, we examined their expression in high‐ and low‐Gleason score PCa specimens. The mRNA expression of *NPM1* and *FBL* was not significantly enhanced in high‐Gleason score PCa in the TCGA PRAD dataset (supplementary material, Figure S1C,D). We further analyzed the protein expression of NPM1 and FBL in the TMAs of different Gleason score PCa specimens, ranging from Gleason score 6 to 10, using IHC with antibodies against NPM1 and FBL. We found that the expression of NPM1 was significantly enhanced in specimens with Gleason scores 7, 8, and 10 compared to Gleason score 6 (Figure 3A). We found a positive trend in enhanced NPM1 expression in Gleason score 9 specimens compared to Gleason score 6, but this difference was not significant (Figure 3A). The expression of FBL was significantly elevated in Gleason score 7 specimens compared to Gleason score 6 specimens (Figure 3B). The expression of FBL showed a positive trend in enhanced expression in specimens with Gleason scores 8, 9, and 10 compared to Gleason score 6 specimens, but these differences were not significant. These results suggest that the expression of NPM1 increases in high‐Gleason score specimens.

### NPM1 and FBL regulate PCa progression

**Figure 4:**
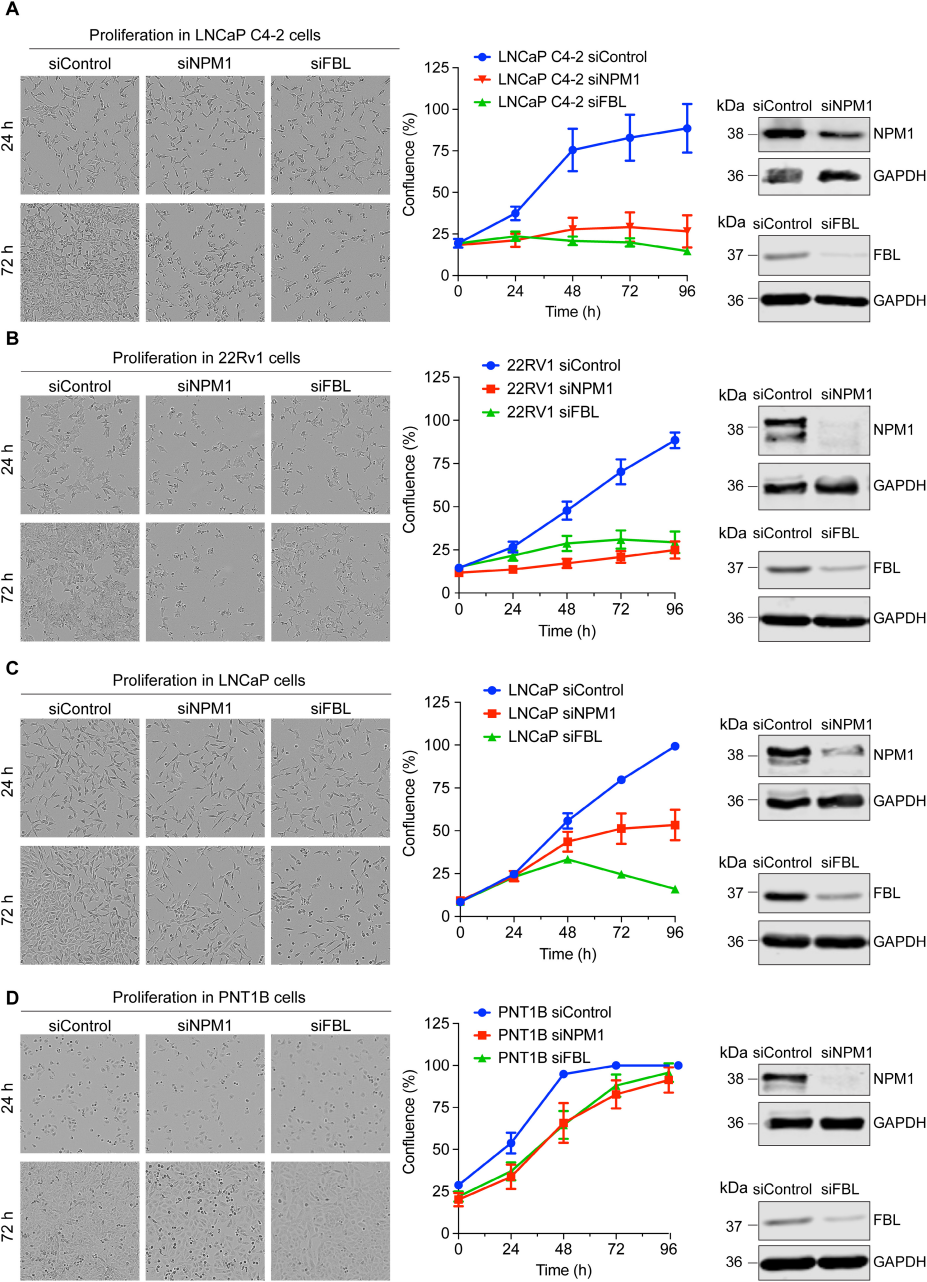
Silencing of NPM1 and FBL reduced the proliferation of PCa cells. NPM1 or FBL were silenced in PCa cell lines, including LNCaP C4‐2 (A), 22Rv1 (B), LNCaP (C), and noncancerous prostatic cell line PNT1B (D) and monitored cell proliferation using Incuyte assay. Phase contrast images corresponding to 24 and 72 h of proliferation are shown in the left panels, and the proliferation curves over 96 h and western blotting showing the efficiency of the silencing of NPM1 and FBL in the respective cell lines are shown in the right panels.

**Figure 5:**
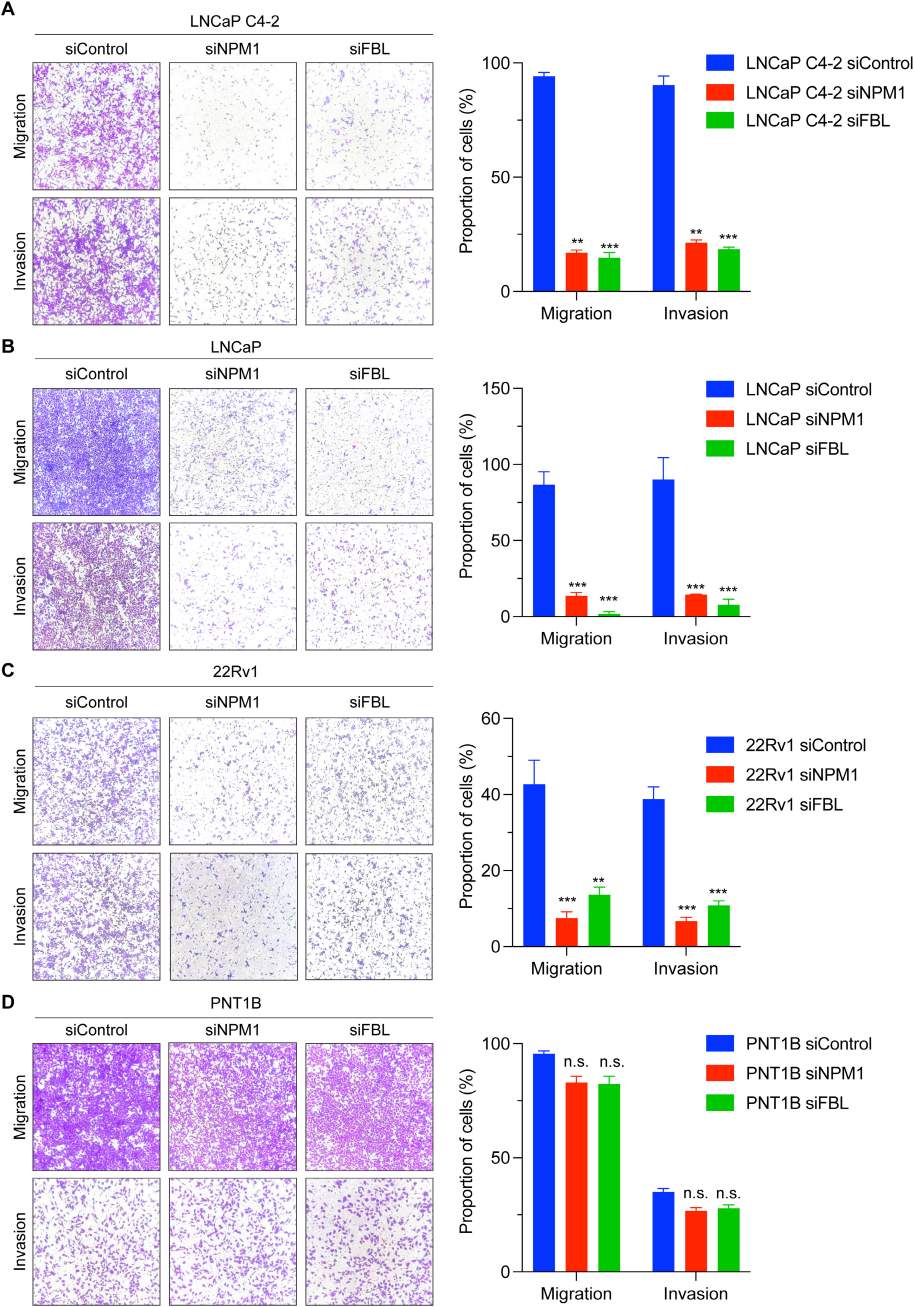
Silencing of NPM1 and FBL reduced the migration and invasion of PCa cells. NPM1 or FBL were silenced in PCa cell lines, including LNCaP C4‐2 (A), LNCaP (B), 22Rv1 (C), and noncancerous prostatic cell line PNT1B (D) cells and measured migration (upper panels) and invasion (lower panels) by Transwell insert assays. Representative images of migrated and invaded cells and their quantification are shown. **p < 0.01; ***p < 0.001; n.s., not significant.

Since NPM1 and FBL are expressed at significantly higher levels in treatment‐resistant and aggressive PCa specimens, we investigated whether they could also support the growth and progression of PCa. We used specific siRNAs (siNPM1 and siFBL) to silence NPM1 and FBL in three PCa cell lines, including LNCaP C4‐2, 22Rv1, and LNCaP, and noncancerous PNT1B cells. The silencing efficiency of NPM1 and FBL was monitored by western blotting (Figure 4A–D) and RT‐qPCR (supplementary material, Figure S3) These cells were then used to assess cell proliferation by Incucyte (Sartorius) and to assess cell migration and invasion using Transwell insert assays, compared to a control (siControl). Our results showed that silencing NPM1 or FBL significantly reduced the proliferation of LNCaP C4‐2, 22Rv1, and LNCaP cells (Figure 4A–C). Silencing of NPM1 and FBL did not significantly affect the proliferation of PNT1B cells compared to PCa cells (Figure 4D). For migration and invasion, we observed a similar trend – silencing of NPM1 or FBL reduced the migration and invasion of LNCaP C4‐2, 22Rv1, and LNCaP cells compared to the control cells (Figure 5A–C). Silencing of NPM1 or FBL did not affect the migration and invasion of PNT1B cells (Figure 5D). Together, these results suggest the dependency of PCa cells on NPM1 or FBL for proliferation, migration, and invasion. Since silencing of NPM1 and FBL did not affect the proliferation, migration, and invasion of PNT1B cells, it indicates that noncancerous cells are not strictly dependent on NPM1 and FBL for their proliferation, migration, and invasion.

### Nucleolar proteins regulate the morphology of nucleoli

**Figure 6:**
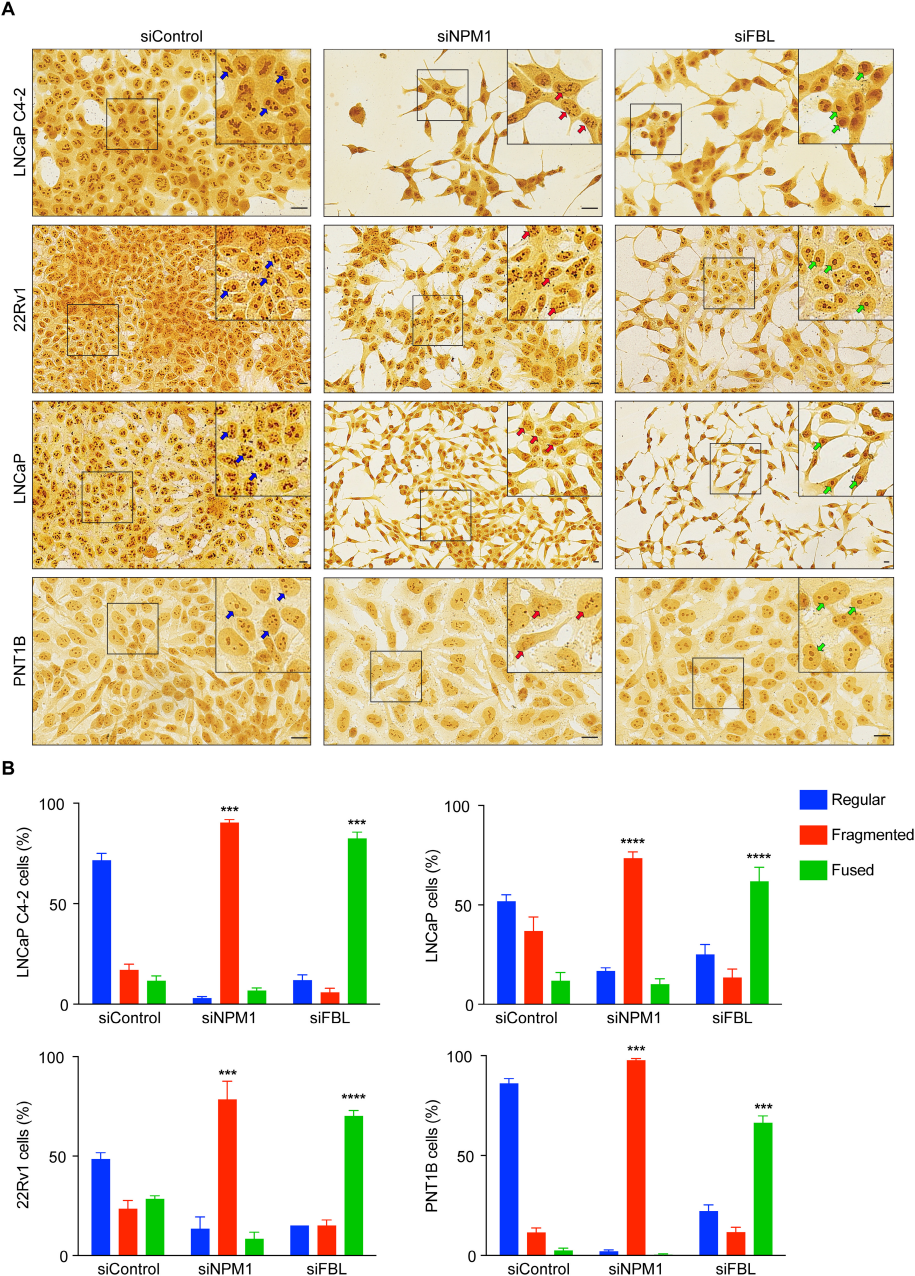
Silencing of NPM1 and FBL changed the morphology of prostate cancer and noncancerous prostatic cells. (A) NPM1 or FBL was silenced in LNCaP C4‐2, 22Rv1, LNCaP, and PNT1B cells, and they were subjected to silver staining. Scale bar, 10 μm. A part of the image is enlarged and shown as an inset. Cells with regular, fragmented, or fused (condensed) morphologies are indicated by arrows. (B) The quantification of nucleoli with regular, fragmented, or fused (condensed) morphologies is shown in the bottom panels. ***p < 0.001; ****p < 0.0001.

To determine if NPM1 and FBL play roles in regulating the morphology of the nucleolus in PCa cells, we silenced them using siRNAs in LNCaP C4‐2, 22Rv1, and LNCaP cells and analyzed their morphology by silver nitrate staining. Silver nitrate stains argyrophilic proteins in the nucleolus, making the nucleolus appear a dark brown color, which is visible by light microscopy [43, 44]. We found that the silencing of nucleolar proteins altered the morphology of nucleoli in LNCaP C4‐2, 22Rv1, and LNCaP cells. The nucleoli appeared dispersed and irregular in siControl cells, while they became fragmented in siNPM1 cells (Figure 6A,B). On the other hand, the nucleoli became fused and condensed in siFBL cells compared to siControl cells (Figure 6A,B). To determine if NPM1 and FBL similarly regulated nucleolar morphology in noncancerous cells, we silenced them in normal prostatic PNT1B cells and monitored nucleolar morphology. Interestingly, we found that silencing NPM1 fragmented the nucleoli while silencing of FBL fused and condensed the nucleoli (Figure 6A,B). These results show that FBL and NPM1 are regulators of nucleolar morphology in both PCa and noncancerous prostatic cells.

## Discussion

We investigated the roles of two nucleolar proteins, NPM1 and FBL, in PCa. Our study found that NPM1 and FBL were localized in the GC and DFC regions of the nucleolus across various PCa and noncancerous prostatic cell lines. We observed a significantly higher expression of NPM1 and FBL in aggressive CRPC and NEPC patient specimens. Additionally, our analysis revealed that PCa specimens with a high Gleason score exhibited higher expression levels of NPM1 compared to those with a low Gleason score. Silencing of NPM1 or FBL led to decreased cell proliferation, migration, and invasion in PCa cells, with no significant effect on noncancerous cells. Furthermore, silencing NPM1 fragmented nucleoli while silencing FBL condensed nucleoli in both PCa and noncancerous prostatic cells. Our results suggest that the nucleolar expression of NPM1 and FBL is elevated in aggressive forms of PCa and that these proteins play a crucial role in regulating nucleolar morphology and PCa progression.

NPM1 is a prominent phosphoprotein located in the GC region of the nucleolus. It plays an essential role in regulating ribosome maturation and nuclear export of ribosome subunits to the cytoplasm [45]. Our research indicates that NPM1 levels are significantly elevated in CRPC, NEPC, and high‐Gleason score PCa. Our finding is consistent with higher NPM1 expression observed in various cancer types, including prostate adenocarcinoma, lung adenocarcinoma, colorectal cancer, pancreatic ductal adenocarcinoma, breast cancer, and bladder cancer. In prostate adenocarcinoma, higher expression of NPM1 was observed in Gleason 4 pattern specimens compared to Gleason 3 pattern specimens [46]. In lung adenocarcinoma, increased NPM1 expression is associated with poor patient prognosis [47]. Similarly, elevated NPM1 levels are linked to lymph node metastasis and reduced survival rates in patients with colorectal cancer [48]. Furthermore, NPM1 expression is heightened in triple‐negative breast cancer [49]. In bladder cancer, NPM1 contributes to tumor development and is correlated with poor patient prognosis, larger tumor size, advanced tumor stage, and increased recurrence rates [50].

We observed a significant reduction in proliferation, migration, and invasion in PCa cells when NPM1 was silenced, while these effects were not observed in noncancerous prostatic cells. Our results suggest that NPM1 may positively regulate the progression of PCa. Previous studies have identified NPM1's role in regulating cell growth, proliferation, and transformation [51]. NPM1 also plays a critical role in maintaining genetic stability by regulating centrosome duplication. Consequently, the overexpression of NPM1 is associated with cellular transformation and uncontrolled cell growth [52]. Additionally, the expression of NPM1 increases in response to mitogenic stimuli, and higher levels of NPM1 are detected in highly proliferating and malignant cells [53,54]. Silencing NPM1 has been shown to reduce cancer growth. For instance, inhibiting NPM1 reduces the proliferation, migration, and invasion of lung adenocarcinoma cells [47] and colon cancer cells [48]. Furthermore, NPM1 silencing can impede cell cycle progression in breast cancer cells [55].

NPM1 is frequently mutated and rearranged in tumors, particularly in acute myeloid leukemia (AML), where it is altered in about one‐third of patients. This makes NPM1 the most commonly altered gene in hematopoietic cancers. Its mutations are being developed as a diagnostic tool for AML [56]. Given the protumoral functions of NPM1 across various cancer types, it holds significant potential as a therapeutic target. Currently, inhibitors aimed at blocking NPM1 in cancer cells are under development [57, 58]. Our findings indicate that NPM1 is expressed at higher levels in aggressive PCa. Our observations on a higher NPM1 expression in aggressive PCa and reduced proliferation, migration, and invasion of PCa cells after NPM1 silencing suggest that targeting NPM1 could be a promising therapeutic strategy to inhibit PCa progression.

Like NPM1, FBL is an abundant nucleolar protein [59]. FBL exhibits several functional features that are essential for ribosome biogenesis. It regulates ribosomal DNA (rDNA) synthesis and pre‐rRNA cleavage [60, 61]. FBL has methyl transferase activity, catalyzing the rRNA 2’‐O‐ribose methylation (2’‐O‐Me) [62]. The higher expression of FBL detected in CRPC and NEPC specimens indicates that FBL is a marker of aggressiveness in PCa. Previous studies have linked the expression of FBL to cancer [63, 64]. Enhanced expression of FBL leads to distinct rRNA 2’‐O‐Me modification patterns in cancer cells, producing more heterogeneous ribosomes and thus directly affecting the translational activities of ribosomes, leading to alteration in the translation of specific mRNAs encoding oncogenic proteins [65, 66]. The expression of FBL is upregulated in several human cancers, including hepatocellular carcinoma [67] and esophageal squamous cell carcinoma [68]. High FBL expression is associated with larger tumor diameter, advanced tumor stage and a poor prognosis in hepatocellular carcinoma [67]. An omics study identified enhanced FBL expression in esophageal squamous cell carcinoma and is associated with poor survival of patients [68].

Since FBL silencing reduced PCa cells' proliferation, migration, and invasion without affecting normal prostatic cells, our results suggest that FBL supports PCa progression. Previous studies identified FBL as a cancer‐promoting factor. FBL is required for proliferation and clonogenic survival in PCa cells [19]. FBL promotes cellular proliferation, colony formation, and resistance to doxorubicin in breast cancer cells [69, 70]. In hepatocellular carcinoma, elevated FBL expression correlates with increased cell proliferation and enhanced metastatic potential. Silencing of FBL in hepatocellular carcinoma cells leads to reduced proliferation, migration, and invasion [67, 71]. These findings underscore FBL's critical function in cancer progression, making it a potential target for therapeutic interventions across multiple cancer types.

We found a significant change in the morphology of nucleoli in NPM1‐ and FBL‐silenced cells. The nucleoli are irregular and dispersed in control PCa cells. The nucleoli appeared fragmented in NPM1‐silenced cells, while they appeared condensed in FBL‐silenced cells. The nucleolar formation is explained based on the liquid–liquid phase separation (LLPS) of nucleolar proteins [13, 72]. NPM1 and FBL are intrinsically disordered proteins with the ability to undergo LLPS [59, 73]. NPM1 forms large oligomers and binds RNA or ribosomal proteins to undergo LLPS [24]. LLPS facilitates NPM1 to create a scaffold that supports the assembly of ribosomes in the GC region of the nucleolus [74, 75]. In the absence of NPM1, the NPM1‐assisted scaffold for the assembly of ribosomes will not be possible. The fragmented nucleolar morphology observed in NPM1‐silenced cells could be due to the failure to form an NPM1 scaffold for supporting the assembly of ribosomes in the GC region.

The LLPS of FBL drives the assembly of DFC that supports pre‐rRNA sorting [59]. FBL diffuses to the DFC, where its glycine‐ and arginine‐rich (GAR) domain facilitates local self‐association, forming phase‐separated clusters. This process immobilizes FBL‐interacting pre‐rRNA, promoting the directional transport of nascent pre‐rRNA while aiding in pre‐rRNA processing and DFC formation. In cells with silenced FBL, we have observed condensed nucleoli. The DFC connects the FC with the GC region [76]. A single nucleolus can contain multiple DFCs. The defect in DFC formation in FBL‐silenced cells could potentially hinder the connection between the FC and GC regions. This disruption could lead to the loss of normal structural architecture in nucleoli, resulting in nucleolar condensation in FBL‐silenced cells.

Interestingly, the silencing of NPM1 and FBL in normal prostatic PNT1B cells also changed the nucleolar morphology. NPM1 silencing condensed the nucleoli, while FBL silencing fragmented the nucleoli, albeit to a lesser extent than PCa cells. Despite a change in nucleolar morphology, PNT1B cells were not significantly affected in their proliferation, migration, and invasion with NPM1 or FBL silencing. This indicates that PCa cells are dependent on NPM1 and FBL for cancer progression than noncancerous cells. Further studies are needed to understand why PCa cells exhibit a dependency on NPM1 and FBL.

## Conclusion

Our results indicate that elevated NPM1 and FBL are associated with aggressiveness in PCa. Their expression is significantly higher in CRPC and NEPC compared to HNPC. An elevated level of NPM1 is also found in high‐Gleason score PCa. Silencing NPM1 or FBL changed nucleolar morphology and decreased proliferation, migration, and invasion in PCa. The elevated expression of NPM1 and FBL in aggressive PCa and their role in PCa progression indicates a potential to develop them as markers of aggressiveness and therapeutic targets for the early detection and treatment of aggressive PCa.

## Author Contributions

SS (Saffarain), ZC and SS (Somasekharan) designed the experiments. SS (Somasekharan) supervised the study. SS (Saffarain), ZC and JL conducted the experiments. SS (Saffarain), ZC, HZO and SS (Somasekharan) performed data analysis and interpretation. SS (Saffarain), ZC, JL and SS (Somasekharan) wrote the manuscript. All authors have read and agreed to the published version of the manuscript.

## Supporting information


**Figure S1.** In silico analysis of NPM1 and FBL in prostate adenocarcinoma (PRAD) TCGA dataset
**Figure S2.** Expression of NPM1 and FBL in benign prostatic hyperplasia (BPH) specimens
**Figure S3.** Analysis of mRNA expression of NPM1 and FBL

## Data Availability

The data that support the findings of this study are available from the corresponding author upon reasonable request.
