# Supplementary material for: Elevated NPM1 and FBL expression correlates with prostate cancer aggressiveness and progression
Source: J Pathol. 2025 Jul 24;267(1):56–68. doi: 10.1002/path.6447 (PMC12337817; doi:10.1002/path.6447)
Supplement: Supplementary file 1 — Figure S1. In silico analysis of NPM1 and FBL in prostate adenocarcinoma (PRAD) TCGA dataset Figure S2. Expression of NPM1 and FBL in benign prostatic hyperplasia (BPH) specimens Figure S3. Analysis of mRNA expression of NPM1 and FBL [file PATH-267-56-s001.docx]

**Elevated NPM1 and FBL expression correlates with prostate cancer aggressiveness and progression**

S Saffarian, Z Cai *et al.* *J Pathol* <https://doi.org/10.1002/path.6447>

**Supplementary Figures S1–S3**


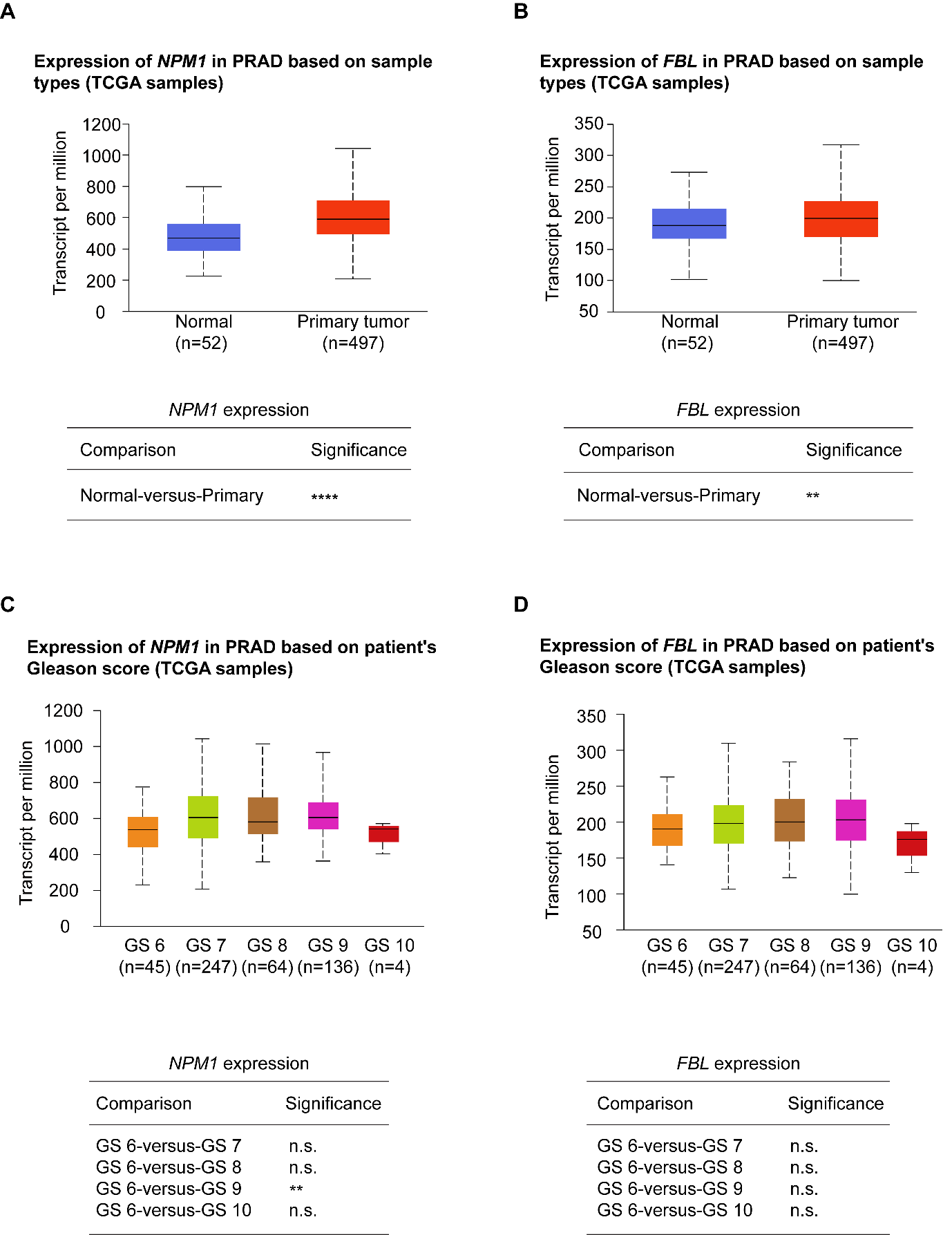


**Figure S1.** **In silico analysis of *NPM1* and *FBL* in prostate adenocarcinoma (PRAD) TCGA dataset.** Expression of NPM1 (A) and FBL (B) in normal and PCa specimens. Expression of *NPM1* (C) and *FBL* (D) in different Gleason score PCa specimens. ***p* < 0.001; n.s., not significant.


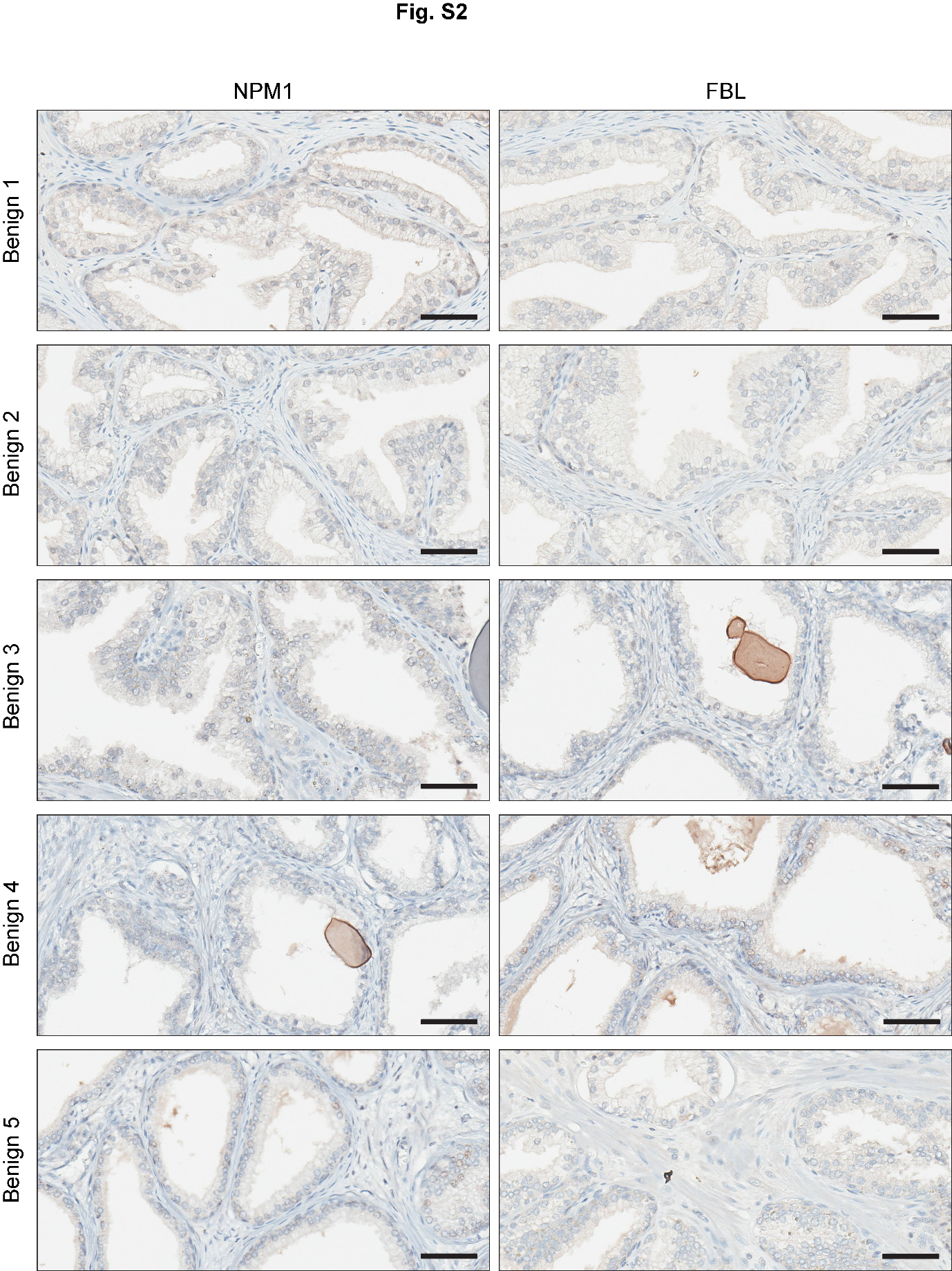


**Figure S2. Expression of NPM1 and FBL in benign prostatic hyperplasia (BPH) specimens.** BPH specimens were IHC stained with anti-NPM1 (left panel) and anti-FBL antibodies (right panel). Scale bar, 60 μm.


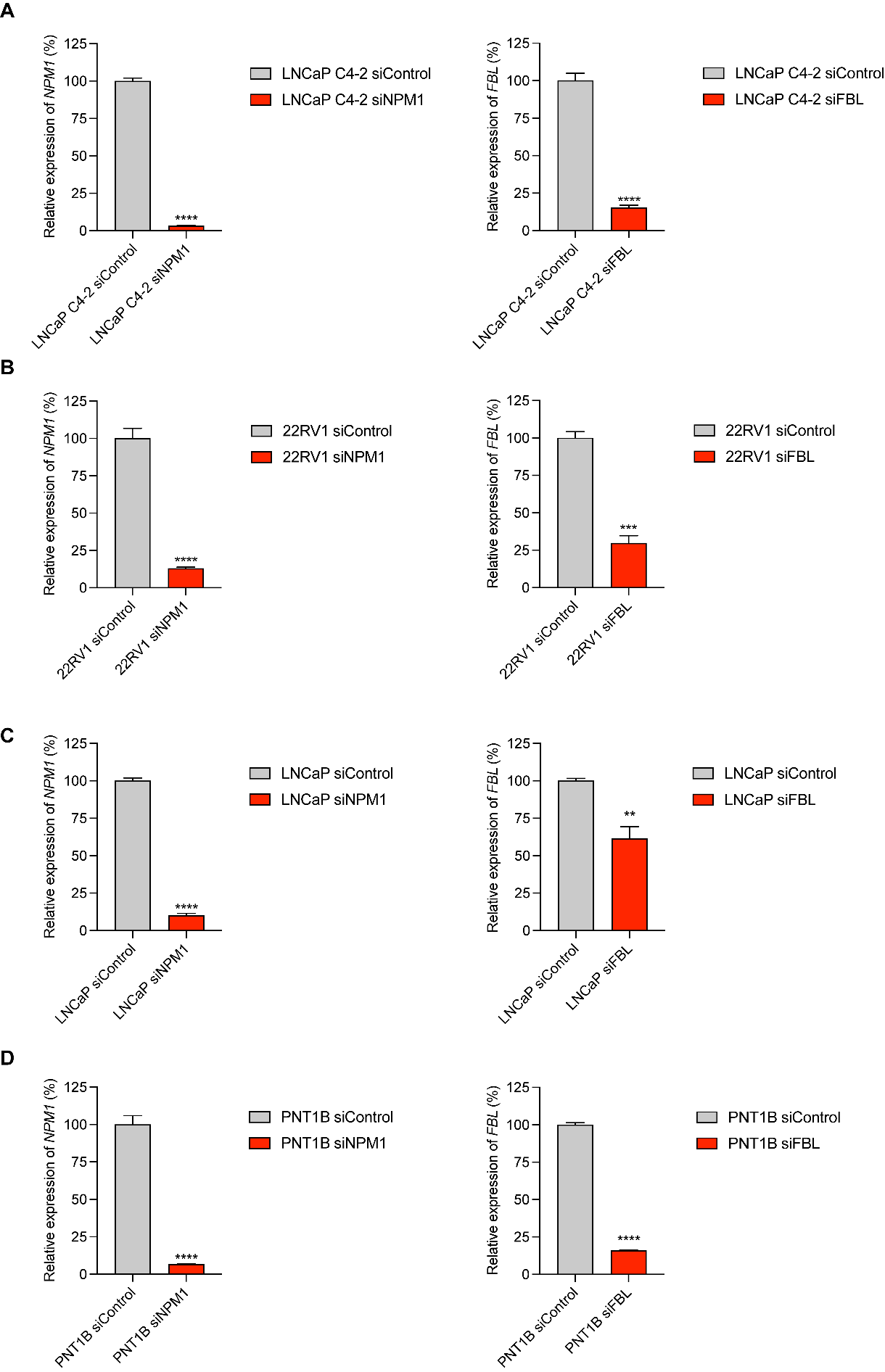


**Figure S3.** **Analysis of mRNA expression of *NPM1* and *FBL*.** Expression of *NPM1* and *FBL* as analyzed by RT-qPCR in (A) LNCaP C4-2 (B) 22Rv1, (C) LNCaP, and (D) PNT1B cells. ***p* < 0.01; ****p* < 0.001; *****p* < 0.0001.
